# Thyroid-like follicular carcinoma of the kidney presenting on a ten year-old prepubertal girl

**DOI:** 10.1590/S1677-5538.IBJU.2018.0471

**Published:** 2019-09-02

**Authors:** Lisieux Eyer de Jesus, Celine Fulgêncio, Thais Leve, Samuel Dekermacher

**Affiliations:** 1 Departamento de Cirurgia e Urologia Pediátrica, Hospital Federal dos Servidores do Estado, Rio de Janeiro, RJ, Brasil

**Keywords:** Kidney Neoplasms, Carcinoma, Pediatrics

## Abstract

The very rare thyroid-like carcinoma of the kidney (TLCK) is microscopically similar to thyroid follicular cell carcinoma (TFCC). Differential diagnosis with secondary thyroid tumors depends on non-reactivity to immunohistochemical (IHC) markers for TFCC (thyroglobulin - TG and TTF1). We herein describe the fourth Pediatric case in literature and extensively review the subject. Only 29 cases were published to the moment. Most cases were asymptomatic and incidentally detected. Most tumors are hyperechoic and hyperdense with low grade heterogenous enhancement on CT and MRI. Most patients were treated with radical nephrectomy, but partial nephrectomy was used in some cases, apparently with the same results. Metastases are uncommon and apparently do not change prognosis, but follow-ups are limited. Up to the moment, TLCK presents as a low grade malignancy that may be treated exclusively with surgery and frequently with partial kidney renal preservation. A preoperative percutaneous biopsy is a common procedure to investigate atypical tumors in childhood and adult tumors. To recognize the possibility of TLCK is fundamental to avoid unnecessary thyroidectomies in those patients, supposing a primary thyroid tumor.

## INTRODUCTION

Thyroid-like carcinoma of the kidney (TLCK) is microscopically similar to thyroid follicular cell carcinoma (TFCC) and depends on non-reactivity to immunohistochemical (IHC) markers for TFCC (thyroglobulin-TG and TTF1) and on exclusion of other primary renal tumors for diagnosis. We describe a Pediatric case with a literature review.

## CLINICAL SCENARIO AND RATIONAL

### Clinical and pathologic findings

**Figure 1:**
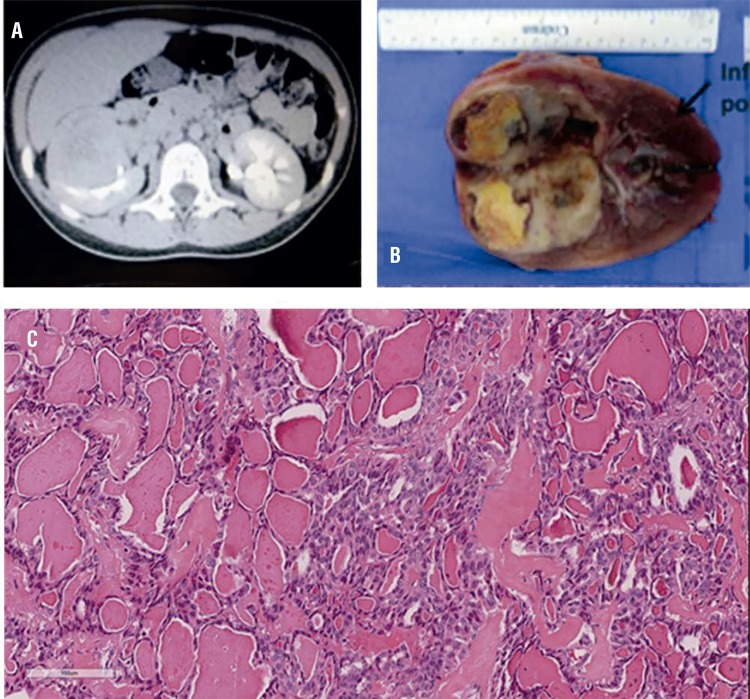
Thyroid-like carcinoma of the kidney. (A) CT with contrast, showing heterogeneous low grade enhancement of the tumor. (B) Well defined tumor showing cystic, hemorrhagic and solid areas. (C) Follicular architecture with microfollicles and macrofollicles filled with colloid-like material.

A 10 year-old pre-pubertal female presented with abdominal pain and occasional nausea and vomiting for two months. Her physical examination was normal. An abdominal ultrasound showed an encapsulated 82x69x42mm solid mass with mixed echogenicity, predominantly hyperechoic, in the medium/superior poles of the right kidney. A thoracoabdominal CT confirmed an exophytic, lobulated, solid, circumscribed 63x60x47mm mass in the superior and anterior medium third of the right kidney, with heterogeneous low enhancement after contrast injection. Necrotic and cystic areas were present, abutting but not invading the renal pelvis and hilar vessels. Augmented perihilar and pericaval lymph nodes were present (Figure-1). Her thyroid was functionally normal. No thyroid, ovarian, pelvic, cervical or thoracic tumors were demonstrated.

Right radical nephrectomy and locoregional lymphadenectomy were performed. The kidney mass measured 55x50x50mm and was well encapsulated, showing cystic and solid areas (Figure-1). Microscopically the main feature was the strong resemblance to thyroid tissue. At low magnification the tumor was composed of follicles of variable size. The follicles were lined by cuboidal or flattened epithelial cells and the material in the follicles was eosinophilic. The nuclei were round with uniform distribution of chromatin. Calcification, fibrosis, hemorrhage and necrosis were focally seen. The mass was restricted to the kidney, without vascular, adrenal, renal sinus or lymphatic invasion. The lymph nodes showed no metastases. Immunohistochemistry demonstrated non-reactivity for TTF-1 and thyroglobulin. The tumor cells were also non-immunoreactive with CK20, CD117 and RCC. Other markers were tested with positive results for PAX8, CK7, EMA and vimentin. After 1 year 7 months the patient persists asymptomatic with normal abdominal ultrasounds.

## DISCUSSION AND FUTURE PERSPECTIVES

Twenty-five cases of TLCK have been described. Another 4 are available in Chinese (n=3) and German (n=1). Females predominate (14 females, 6 males, 1 unknown). Ages vary between 19-83 years-old (mean 42.7, median 35). Females tended to be younger (median 32 versus 55 years-old for males). Only 3 other pediatric cases were published (5.3-14 years-old, 2/3 females) (1).

Most cases were asymptomatic, incidentally detected. Approximately 1/5 presented hematuria and/or flank pain. One patient each showed weight loss, anemia and hypertension (cured after resection of a perihilar tumor) (2). Many tumors were associated with previous malignancy (5/22) or pre-neoplastic conditions (1 case, adult polycystic renal disease).

TLCK predominates in the right kidney (14/22 cases, 63.6%), maximal dimensions varying between 11 and 118mm (mean 44.8mm). Four (18.2%), 11 (50%) and 7 (31.8%) affected the upper, mid and lower poles, respectively.

Most tumors were hyperechoic (contrasting with TFCC, usually hypoechoic) and hyperdense with low grade heterogenous enhancement on CT and MRI. On pre-contrast MRI TLCK showed high signal on T1 and low signal on T2, as compared to the kidney parenchyma (3). Some presented calcifications. No vascular or urothelial invasion were described, but vessels and renal calices might be displaced. Only two PET scan results are available, both positive to FDG marking (3, 4). Abdominal lymph nodes augmentation most commonly did not correspond to metastasis.

Most patients were treated with radical nephrectomy. Partial nephrectomy was used in 6 cases, apparently with the same results. Three patients presented lung metastases (3, 5). Curiously, in one case the metastatic nodule was immunoreactive to TTF1, as opposed to the kidney specimen (6). Three adults showed abdominal lymph node metastasis (5, 7).

Follow-up is limited (median 20 months). Most patients did not show progression of the disease or metastases (Table-1).

**Table 1:** Summary of clinical characteristics of the tumors described in the literature plus present case.

Author/publication year	Age (years)/sex	Presentation	Past history	Local/size (mm)	Imaging	Nephrectomy (T/P)/FU
Alesssandrini, 2012 (16)	76 M	Hematuria	Prostate cancer	L UP, 50	(CT) Hyperdense, well vascularized, necrotic center, extension to adipose tissue, enlarged lymph nodes (no metastases).	T/ 11 months NED
	41 F	Incidental	Hodgkin lymphoma	R LP, 50	(CT) complex cystic, hyperdense no enhancement. (MRI) solid septa	P/ 4 months NED
Muscara 2017 (12)	27 M	Incidental	-	Left UP, 65	-	P/8 months NED
Amin 2009 (7)	N=6 (29-83), 3M 3 F	All incidental	1 Colon cancer, 1 osteosarcoma	5R 1L / 1 UP, 4 MP, 2 LP/ 19-40	-	6 T/ 7-84 months, NED
Dawane 2015 (17)	49 F	Incidental	-	L MP 24	(CT) contrast enhancement, extension to adipose tissue.	P/ 5 years, NED
Khoja 2014 (18)	31 F	Hematuria, weight loss, flank pain (3 years), anemia	Normal thyroid (I/F), normal ovaries (I)	L UP 43	(CT) heterogeneous enhancing, distorting collecting system, lymph node enlargement (no metastases).	T/ 21 months NED
Jung 2006 (13)	32 F	Incidental	Normal thyroid (I/F), normal ovaries (I)	R LP/ 118	(CT) contrast enhancing, hydronephrosis.	T/ 6 months NED
Dhillon 2011 (5)	34 F	Hematuria, flank pain	Normal thyroid (I/F)	R MP/ 63	Multiple pulmonary nodules (biopsy “thyroid carcinoma”)	“systemic treatment” for thyroid cancer (1 year) + T nephrectomy/ 3 months NED
Lin 2014 (8)	65 M	Hematuria (4 years), back pain (1 week)	Normal thyroid (I/F)	R MP, 80	Hypoechogenic hilar mass, (CT) “renal carcinoma”, normal fascia/lymph nodes.	T/ 2 years NED
	59 F	Incidental	Normal thyroid (I/F)	R MP, 60	Normal fascia/lymph nodes.	T/ 1 month NED
Wu 2014 (4)	19 F	Incidental	Leukemia (5 years-old)	R LP 28	(CT) heterogeneous hyperdense. No lymph nodes. No metastasis. PET (+).	Biopsy + P nephrectomy/ 21 months NED.
Wang 2017 (2)	25 F	Severe hypertension (normal post-operative)	Normal thyroid (I/F), normal ovaries (I)	R MP 30	(CT) inhomogenous enhancement, calcifications. Ovaries normal (imaging).	P/ 2 years NED.
Ghaouti 2014 (10)	68 F	Incidental	Normal thyroid (I/F), normal ovaries (I)	R MP 11	(MRI) Cystic, no enhancement	P/ no FU reported
Volavsek 2013 (11)	34 ?	Incidental	Nephrolithiasis,polycystic disease, adult type.	L LP 50 mm	Hyperechogenic cyst	T/ 6 months NED.
Sterlacci 2008 (6)	28 F	Incidental		L MP 44	(CT) Heterogeneous, no capsule infiltration, displacement of blood vessels. Left lung nodule.	Thyroidectomy (presumed metastatic thyroid cancer, despite normal imaging). Lung lumpectomy. T nephrectomy/ 5 years NED.
Vicens 2014 (3)	24 F	Hematuria, flank pain		R MP, 60	(CT) displaying calices, bilobulated, peripheral calcification, hyperdense, low grade enhancement, peak on delayed phase. Multiple pulmonary nodules, enlarged abdominal lymphonodes. (MRI) increased signal T1, low signal T2, low grade enhancement. PET scan: mild FDG uptake.	T/post op therapy with sunitinib for lung metastases. No FU data.
Malde 2013 (19)	29 F	Flank pain	Thyroid normal (F)	L LP, 58	(CT) Complex multiseptated partially cystic, low attenuation, no enhancement. (MRI) no enhancement.	T. No FU data.
Our case	10 F	Flank pain, nausea, vomiting	Thyroid (I/F) and ovaries I) normal	R +SP/MP, 63	US hyperchoic heterogeneous, CT exophytic anterior superior/medium pole, heterogeneous enhancement, necrotic and cystic areas, lymph node enhancement (no metastases)	T. NED, normal imaging after 19 months.

**NED** = no evidence of disease; **FU** = follow up; **F** = female; **M** = male; **Fu** = function; **I** = imaging; **UP** = upper pole; **MP** = mid pole; **LP** = lower pole; **L** = left; **R** = right; **T** = total; **P** =partial

Differential diagnosis depends on IHC profile. The diseases to be considered are:

### 1.Malignancies:

- Renal metastasis from TFCC from normal or ectopic thyroid tissue (possible on the neck and/or thorax, mainly in the vicinity of the thyroid gland, but not in kidney tissue (6)). Less than 40 cases were described (4), generally associated with widespread metastatic disease (mostly to the lungs, lymph nodes and bones). The metastases are positive to TTF1/TG. A primary tumor should be detectable.
- Metastasis from struma ovarii (2% of the ovarian tumors, malignant in 5-10% of the cases). Metastases are rare (5%), preferentially to the liver and peritoneum, and positive to TTF1/TG (6).
- Papillary renal cell carcinoma may show patchy “thyroid-like” areas, but the typical architecture usually predominates. IHC is positive to kidney tumor markers.
- Renal carcinoids may show zonal “follicular” architecture, positive to neuroendocrine markers (synaptophysin, CD 56 and chromogranin). Oncocytomas and metanephric adenomas may also show focal or patchy “follicular” architecture, due to eosinophilic intraluminal deposits in areas of tubular differentiation.

### 2.Benign entities:

- Kidney “thyreodization” associated to end-stage kidney disease/pyelonephritis, caused by the deposit of colloid-like protein material in the lumina of atrophic distal tubules/collecting ducts. This is a diffuse and bilateral process, not associated with tumors.

TLCK are well circumscribed, yellow/whitish. Hemorrhagic and necrotic areas are common and may present as intratumoral “cysts” (2, 8, 9). Histologically there are macro and microfollicles filled with amorphous eosinophilic colloid-like material (5, 10), similar to TFCC. The follicles are lined with cuboidal cells with scant eosinophilic cytoplasm, round/oval nuclei and evenly distributed chromatin. Mitotic activity is absent or scarce. There may be focal areas of papillary differentiation, patchy lymphoid aggregates, calcifications (2, 3, 10-12) and cholesterol crystals (12). Fibrous septa presenting muscle and a fibrous pseudocapsule have been described (7, 12). No cases presented with vascular or collecting system invasion. Capsular invasion was seen in two cases (11).

The tumor is, by definition, negative to TG/TTF1, positive to epithelial markers (cytokeratyns AE1/AE3, 7, PAX 2 and 8, Vimentin and EMA) and negative to renal tumor markers (WT1, RCC, CD10).

TLCK was described in 2006 (13), is an emerging entity and has not yet been included in the WHO classification of tumors (14). A possible previous case was positive for thyroid IHC markers (15) and is questionable. The predominance of young women suggests some hormonal influence and the relatively high incidence of previous malignancies suggests that previous treatments and/or specific genetic constitutions predispose to TLCK.

Extra-renal extra-thyroid tumors (cholangiocarcinoma, breast and urothelial carcinomas, endolymphatic sac tumor, plasmacytoma, papillary renal cell carcinoma) may also present “follicular” architecture and are negative for TG and TTF1 (4, 7, 16). Tubular deposits of Tamm-Horsfall glicoprotein are probably the explanation for the colloidal aspect in kidney tumors, including TLCK (5, 10).

Primary thyroid tumors are positive to TTF1/TG, except for poorly differentiated or sarcoma-like malignancies. For kidney tumors, the IHC panel includes vimentin, CK 7, AMACR, CCR and CD10 and WT1 in atypical tumors or children. A ”thyroid tumor” on a kidney specimen is unexpected and at least one patient was, quite understandably, submitted to a thyroidectomy with the presumed diagnosis of metastatic TFCC, despite normal thyroid imaging (6). Non-reactivity to TG/TTF1 and no primary tumor are the clues to avoid this. Table-2 summarizes the IHC profiles and differential diagnosis for TFCC, kidney tumors and TLCK.

**Table 2:** IHC characteristics of TLCK, other kidney tumors and thyroid tumor.

Tumor	Positive	Negative
RCC (Clear cell)	Vimentin	HMWCK
	AE1/AE3	CK7, CK20
	RCCM	e-cadherin,
	PAX2/PAX8	CD117
		AMACR
RCC (papillary)	Vimentin	CD117
	AE1/AE3	
	CK7	
	AMACR	
	RCCM	
	PAX2/PAX8	
Chromophobe RCC/oncocytoma	e-cadherin	Vimentin
	CD117	AMACR
	AE1/AE3	
	CK7 (Chromophobe)	
WT	CD 57 (tubules (+), blastema (-)	CK7
	CK22, CK18, CK8	Myoglobin
	EMA	Chromogranin A
	actin	RCC
	WT1	P53
	desmin	Vimentin - blastema, focally (+)
TFCC	PAX 8	PAX 2
	TTF1, TG, BME1, galactin 3	
TLCK	CK 7, AE1/3	TTF1, TG
	PAX 2, 8	WT1
	Vimentin	RCC
	EMA	CD10
		CEA
		p53

Surgical resection with clear margins is probably curative, despite the limitations of follow-up data. Partial nephrectomy seems to be as successful as total nephrectomy, but the high proportion of mid pole tumors may impose technical difficulties. Metastases are rare and apparently do not compromise the results in most patients. Adjuvant therapy has not been established.
